# Research on the Recognition and Tracking of Group-Housed Pigs’ Posture Based on Edge Computing

**DOI:** 10.3390/s23218952

**Published:** 2023-11-03

**Authors:** Wenwen Zha, Hualong Li, Guodong Wu, Liping Zhang, Weihao Pan, Lichuan Gu, Jun Jiao, Qiang Zhang

**Affiliations:** 1 School of Information and Computer, Anhui Agricultural University, Hefei 230036, China; zhawenwen@stu.ahau.edu.cn (W.Z.); wugd@ahau.edu.cn (G.W.); panweihao17114212@stu.ahau.edu.cn (W.P.); glc@ahau.edu.cn (L.G.); 2 Institute of Intelligent Machines, Chinese Academy of Sciences, Hefei 230031, China; hlli@iim.ac.cn; 3 Institute of Agricultural Economy and Information, Anhui Academy of Agricultural Sciences, Hefei 230031, China; sangold@163.com; 4 Department of Biosystems Engineering, University of Manitoba, Winnipeg, MB R3T 5V6, Canada

**Keywords:** behavior monitoring, target detection, multi-target tracking, edge computing

## Abstract

The existing algorithms for identifying and tracking pigs in barns generally have a large number of parameters, relatively complex networks and a high demand for computational resources, which are not suitable for deployment in embedded-edge nodes on farms. A lightweight multi-objective identification and tracking algorithm based on improved YOLOv5s and DeepSort was developed for group-housed pigs in this study. The identification algorithm was optimized by: (i) using a dilated convolution in the YOLOv5s backbone network to reduce the number of model parameters and computational power requirements; (ii) adding a coordinate attention mechanism to improve the model precision; and (iii) pruning the BN layers to reduce the computational requirements. The optimized identification model was combined with DeepSort to form the final Tracking by Detecting algorithm and ported to a Jetson AGX Xavier edge computing node. The algorithm reduced the model size by 65.3% compared to the original YOLOv5s. The algorithm achieved a recognition precision of 96.6%; a tracking time of 46 ms; and a tracking frame rate of 21.7 FPS, and the precision of the tracking statistics was greater than 90%. The model size and performance met the requirements for stable real-time operation in embedded-edge computing nodes for monitoring group-housed pigs.

## 1. Introduction

In order to identify and track the daily behavior of pigs, this study was carried out. In the last decade, digital technologies have been increasingly used in the livestock sector and farm information management to improve efficiency [1], animal welfare and health, and product quality, and to reduce production costs (labor, feed and energy) and environmental impact. Based on a web search, Gómez et al. [2] identified 83 commercially available technologies for pigs. They also conducted a literature search to identify studies on the validation of sensor technologies for assessing animal-based welfare indicators and found 2463 articles. Of the 10 types of sensors reviewed, cameras were validated in most studies. Computer vision has been widely used for monitoring and assessing animal behavior. One of the key challenges in pig behavior monitoring with automated vision systems is identifying and tracking animals, such as pigs, in groups in real time. Advances in machine learning have provided a powerful tool for pig behavior monitoring based on images, sound, acceleration, etc. For instance, the authors of [3] propose an acceleration sensor capable of identifying prenatal behavior in sows. Another study [4] recommends using pressure sensors to detect sow activity during labor. Cappai et al. used ear tags (ETs) or RFID to record activity during animal production [5]. All sensor devices necessitate direct contact with the pig, and as a result, are vulnerable to unconscious harm and destruction, as well as possible animal welfare and regulatory compliance [6]. Hence, non-contact computer vision methods present advantages. Ref. [7] proposed a behavior recognition algorithm based on Mask R-CNN, which could automatically and effectively detect the mounting behavior of pigs. Ref. [8] used machine vision technologies (threshold segmentation and morphological processing) to realize the non-contact identification of pig drinking. Ref. [9] used a non-invasive method based on an improved YOLOv3 model to identify multiple pigs in a group. Ref. [10] used YOLOv2 and Faster R-CNN to monitor pig postures and drinking behavior. Ref. [11] realized individual pig face recognition by combining the attention mechanism of DenseNet and CBAM. Ref. [12] combined YOLOv5s and ST-GCN algorithms to form a pig behavior recognition system for the effective identification of pig postures and time-series features. Ref. [13] developed a target detection model for weaned piglets based on CenterNet, which was combined with the DeepSort algorithm [14] to achieve multi-target tracking of weaned piglets. Another study [15] utilized deep learning techniques to successfully achieve the automatic recognition of the feeding and foraging behavior of pigs. Liu et al. [16] proposed the use of a CNN+LSTM technique for the spatio-temporal action recognition of tail-biting behavior among pigs within group living environments. Kim et al. [17] implemented a target recognition algorithm within an embedded device to accurately count pigs. Although studies have attempted to utilize deep learning with computer vision to recognize behaviors of animals, previous research shares the following limitations: (i) most algorithms deal with the recognition of postures of individual animals or tracking a single animal, failing to integrate animal posture recognition and tracking into a comprehensive study; and (ii) most models prioritize recognition accuracy but lack optimization for lightweight industrial deployments in the field, which makes them difficult to deploy in small embedded-edge computing nodes for achieving real-time operation.

The objectives of this study were: (1) to develop a multi-objective identification and tracking algorithm based on improved YOLOv5s and DeepSort for group-housed pigs to address the problems of the existing algorithms having a large number of parameters, relatively complex networks and large computational expenditures; and (2) to improve the algorithm with lightweight pruning and port the algorithm to an embedded-edge computing node for the real-time detection and tracking of pigs.

## 2. Materials and Methods

### 2.1. Generation of Datasets for Model Training and Validation

#### 2.1.1. Data Acquisition

**Figure 1:**
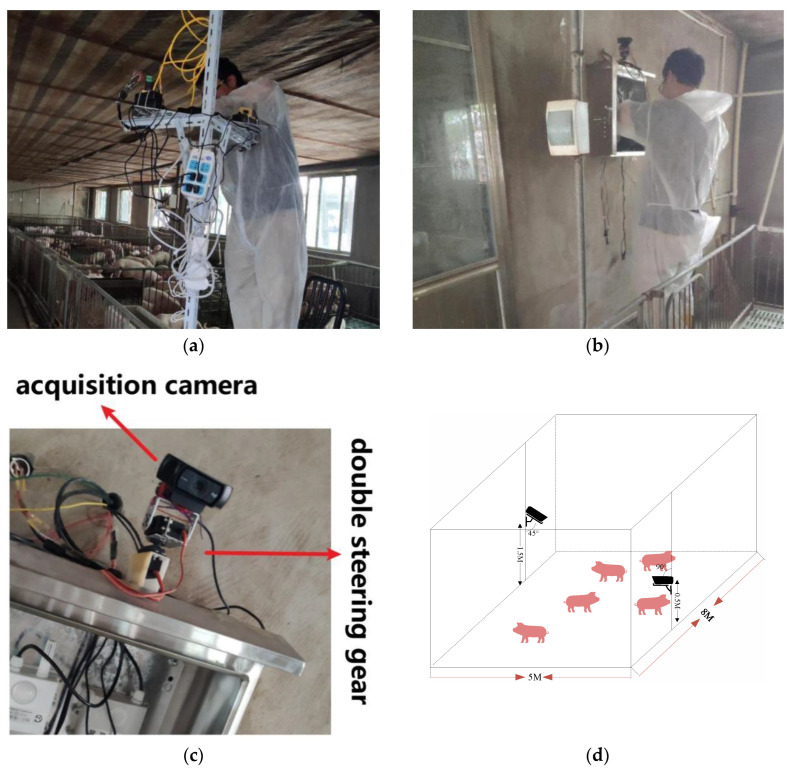
Video capture equipment locations. (**a**) the first set of data-acquisition equipment; (**b**) the second set of data-acquisition equipment; (**c**) details of the second set of data-acquisition equipment; (**d**) 3D spatial presentation of the capture devices. The camera manufacturer is Logitech, the model number is C920 and the location of the camera maker is Shanghai, China.

Datasets were generated from an experiment conducted on a pig farm of Jing Huimeng Agricultural Technology Development Co., Ltd. (Bozhou, China). Pigs were group-housed in the experimental room and two cameras were used to record video images from different angles in different time periods. The first camera and associated data-acquisition and communication equipment were mounted on a pole in a pig pen (Figure 1a) and the image-acquisition system was installed on the wall (Figure 1b). One camera was aimed at the ground at a downward slanting angle of about 45 degrees at a distance of 1.5 m from the ground to obtain top and side information about the target, and the other camera shot was parallel to the ground at a distance of 0.5 m from the ground to obtain side information about the target. The camera angles could be adjusted through a double steering gear unit. The image acquisition platform used a nanoPC-T4 development board as the main control, capturing images at 1080p 30 fps; a series of videos of three and seven adult long white pigs in captivity were captured, and the data streams were sent to the cloud in real time via the RTMP protocol.

A total of 11,000 still images were extracted from the recorded video clips, which were labeled with the LabelImg (version 1.8.6) tool according to the common data annotation format VOC (Visual Object Classes) dataset [18]. Those images were then categorized for four different pig postures: standing, sitting, lying, and lateral (Table 1). In addition to the acquired experimental data, videos from an open source at the University of Nebraska-Lincoln [19] were used to supplement the dataset. In the combined dataset, a total of 28,330 postures were marked, including 8376 standing, 6155 sitting, 6781 lying, and 7018 lateral. 

#### 2.1.2. Data Augmentation

**Figure 2:**
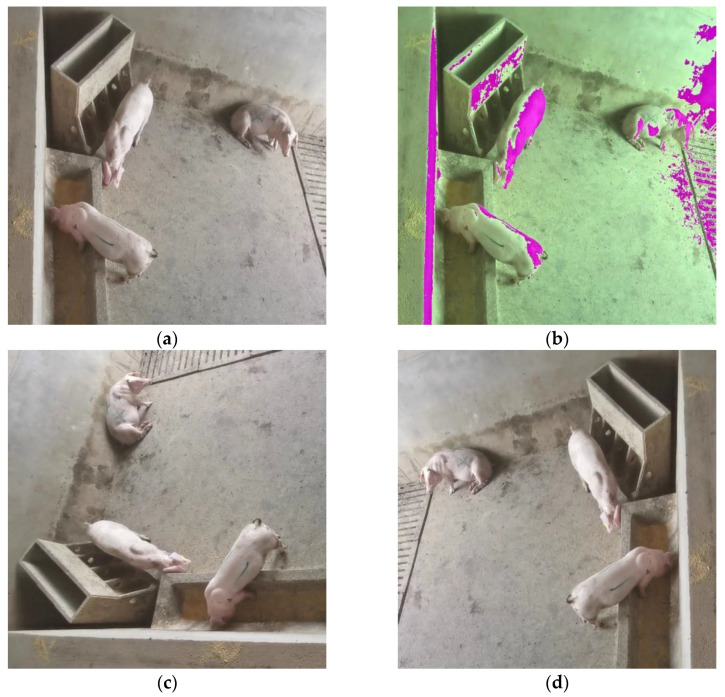
Data augmentation examples. (**a**) Original image; (**b**) Random channel transformed image; (**c**) 90° rotation; (**d**) Left-to-right flipping.

The dataset scale was expanded by using a data augmentation technique: (i) rotating an image for 90°; and (ii) flipping the image (left–right), to improve the generalization ability of the model (Figure 2) [20]. The interference of lighting changes was simulated using the data augmentation technique of random color channel conversion, random saturation conversion, and random chroma conversion [20] to improve the model’s ability to recognize different lighting conditions (Figure 2b). The final dataset was expanded to 65,933 images, which were then divided into a training set and a test set with an 80% to 20% split.

### 2.2. Recognition and Tracking of Pigs

In this study, we use the YOLOv5s algorithm as the basic recognition algorithm to identify the pigs and the DeepSort algorithm to track and process the behavioral gestures of the pigs; due to the large number of parameters of the original YOLOv5 algorithm and DeepSort algorithm and the high energy consumption of the model operation, we make the following improvements: (a) the addition of dilated convolution to the YOLOv5s backbone network to reduce model parameters and computational resource demand while ensuring the receptive field [21]; (b) the addition of a coordinate attention mechanism to observe information from a global perspective and improve the precision of the network mode [22]; (c) pruning the BN layers in the YOLOv5s algorithm backbone network to remove the insignificant channels [23]; and (d) combining with the DeepSort algorithm [13] and porting the model to the embedded-edge computing node.

The YOLOv5s algorithm is a self-updating target recognition algorithm that is widely used in industry and academia, and it outperforms other emerging YOLO algorithms in terms of speed, accuracy, size, and training time. The DeepSort algorithm is a multi-target tracking algorithm that combines deep learning and traditional tracking algorithms to accurately track targets in complex environments. Dilated convolution: dilated convolution is a special convolution operation that inserts zero values around the standard convolution kernel, which enables dilated convolution to reduce the computational amount of convolution while guaranteeing the sensory field. BN layer pruning is a model pruning method that effectively reduces the number of parameters and the computational amount of the model, thus increasing the operational efficiency and inference speed of the model.

#### 2.2.1. Multi-Target Recognition Based on Improved YOLOv5s

**Figure 3:**
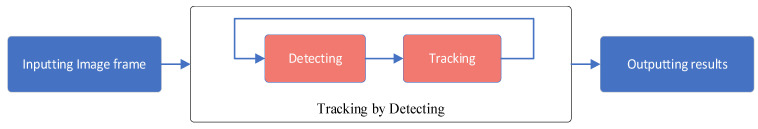
Flowchart of tracking algorithm based on target detection.

The tracking algorithm based on target detection, termed Tracking by Detecting (TbD), consisted of two steps: target detection and multi-target tracking, with the results from the first step being used as the input to the second step (Figure 3).

Detection was based on YOLOv5s, which consisted of Input, Backbone, Neck and Detect. Input was for image adaptive scaling. A mosaic was added to enhance the dataset, and K-means genetic computation was integrated to calculate the optimal anchor frame value of the dataset to improve the detection ability of small targets [24]. The Backbone is mainly responsible for feature extraction of the input image and is composed of the CBS, C3 and SPPF modules. The CBS module is a common basic module in convolutional neural networks, which extracts local spatial information through convolutional operations and normalizes the distribution of feature values through BN layers and finally introduces nonlinear transformation capability through the activation function. The C3 module is composed of three CBS modules, which mainly serve to increase the depth and perceptual field of the network and improve the feature extraction capability. The SPPF module is a pooling module, which aims to achieve the spatial invariance and positional invariance of the input data to improve the recognition capability of the neural network [25]. When the size and location of objects in an image are uncertain, the Neck feature pyramid enables the detection of objects at different scales and different sizes in an image by adding feature layers of different scales to the backbone network. For Detect, Yolov5s adopts the Generalized Intersection Over Union Loss function (GIOU) to evaluate whether the object localization algorithm was precise [25]. The non-maximum suppression algorithm is adopted to remove other prediction results whose contribution value was not the largest, output the classification results with the greatest probability, generate the boundary box, and predict its category.

**Figure 4:**
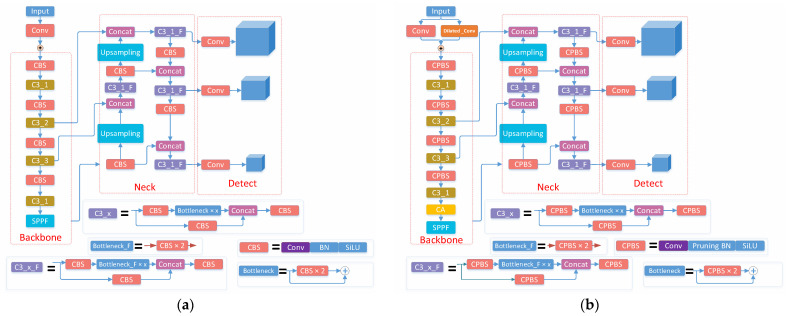
(**a**) Original YOLOv5 structure; (**b**) Schematics of improved YOLOv5s structure.

To make the YOLOv5s detection model lightweight and suitable for deployment to embedded-edge computing nodes, the dilated convolution and attention mechanism were implemented in the YOLOv5s model, and the BN layer was pruned (Figure 4).

**Figure 5:**
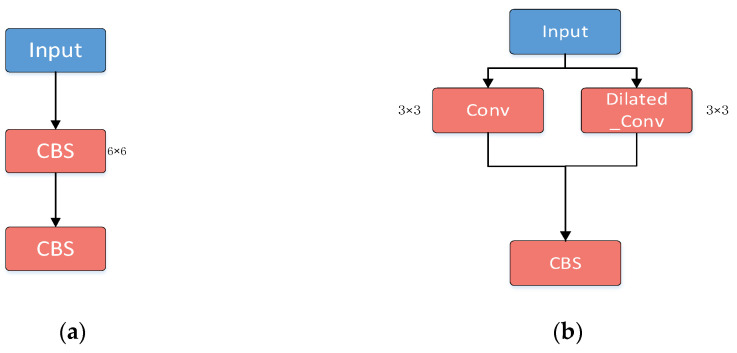
Schematics of dilated convolution addition. (**a**) CBS layer of original YOLOv5s; (**b**) CBS layer of modified YOLOv5s with dilated convolution.

1. Dilated convolution: The 6 × 6 convolution in the first CBS layer at the input of YOLOv5s was replaced with a 3 × 3 ordinary convolution and a 3 × 3 dilated convolution (dilation rate of 2), which was then simplified by removing the subsequent BN layer and SiLU layer (Figure 5). These changes were implemented to reduce the calculation parameters of the input stage by about 50%, and thus the amount of network computation.

**Figure 6:**
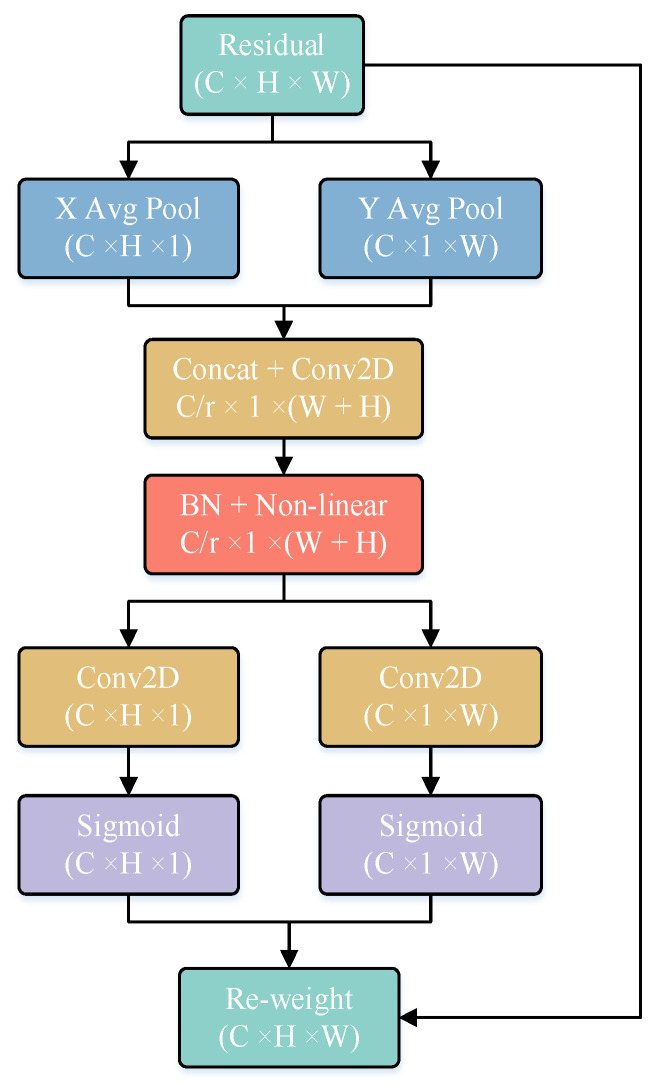
Structure of coordinate attention module.

Coordinate attention: A coordinate attention module proposed by [10] was added to connect the C3_1 layer and the SPPF layer in the backbone network of YOLOv5s (Figure 6). The added CA module optimized the use of limited visual information processing resources, locked the key candidate regions, automatically shielded some background and redundant information, and thus helped the model to better locate and identify the target [26].

**Figure 7:**
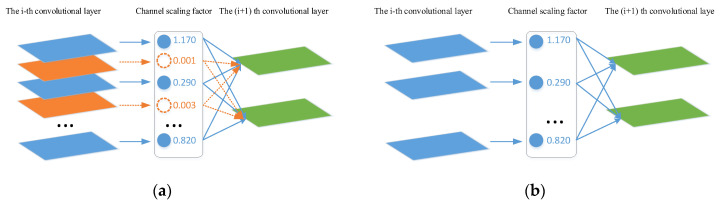
Illustration of pruning BN layers. (**a**) before pruning; (**b**) after pruning.

Pruning the BN layer in the backbone network: The purpose of the BN (Batch Normalization) layer is to speed up the training and convergence of the network, control the gradient explosion to prevent the gradient from disappearing, and prevent the model from overfitting. The BN layer is described by the following equations [23]:

(1) $$\hat{Ζ}=\frac{{Ζ}_{in}-\mu_{B}}{\sqrt{\sigma_{B}^{2}+\varepsilon }}$$

(2) $${Ζ}_{out}=\gamma \hat{Ζ}+\beta$$

where *Z_in_* and *Z_out_* are the input and output of the BN layer, respectively; *B* is the current mini-batch, *µ_B_* and *σ_B_* are the mean and variance of the input activation of *B*, respectively, and *γ* and *β* are the trainable scaling and translation dynamic transformation coefficients, respectively, which can transform the normalization activation into any other scale through a linear transformation. These equations show that the activation size *Z_out_* of each channel of the BN layer is positively correlated with the scaling coefficient *γ*. When *γ* is close to 0, the activation value of *Z_out_* is also minimal. Therefore, removing a channel with *γ* close to 0 would have little effect on the model performance, while computing resource requirements may be reduced. Specifically, after pruning, the resulting network is more compact and requires less runtime memory and computational time. For example, there are five channels in the BN layer, shown in Figure 7, with two channels having _γ_ close to 0, and pruning may reduce the BN layers from five to three.

Pruning was implemented in three steps: sparsification training; prune channels with small scaling factors; and fine-tune the pruned network. The backbone network had a large number of channels per layer output, but not all channels were equally important. To improve [10] the effectiveness of pruning, regularization constraints were added to the loss function by the scaling factor γ for each channel and then multiplied by the output of that channel. This was to jointly train the network weights and these scaling factors and then apply sparse regularization to the latter [27]:

(3) $$Loss=Loss\_0+\lambda \sum_{\gamma \in \Gamma }g\left(\gamma \right)$$

where *Loss*_0 is the original loss function, *g*(*γ*) = |*γ*| is the L1-norm, and *λ* is the regularization coefficient, which was used to balance the two items before and after and could be adjusted according to the dataset (a value of 0.002 was used in this study). By adding regularization constraints, the parameters of the BN layer would be sparse, making it convenient to find those channels with *γ* close to 0 for pruning [23]. Pruning may temporarily degrade the network performance, but this effect can be compensated for by the subsequent fine-tuning of the pruned network, i.e., pruning iterations were performed and model performance was judged to finally obtain a model with the best performance and smallest number of channels. 

#### 2.2.2. DeepSort Tracking

**Figure 8:**
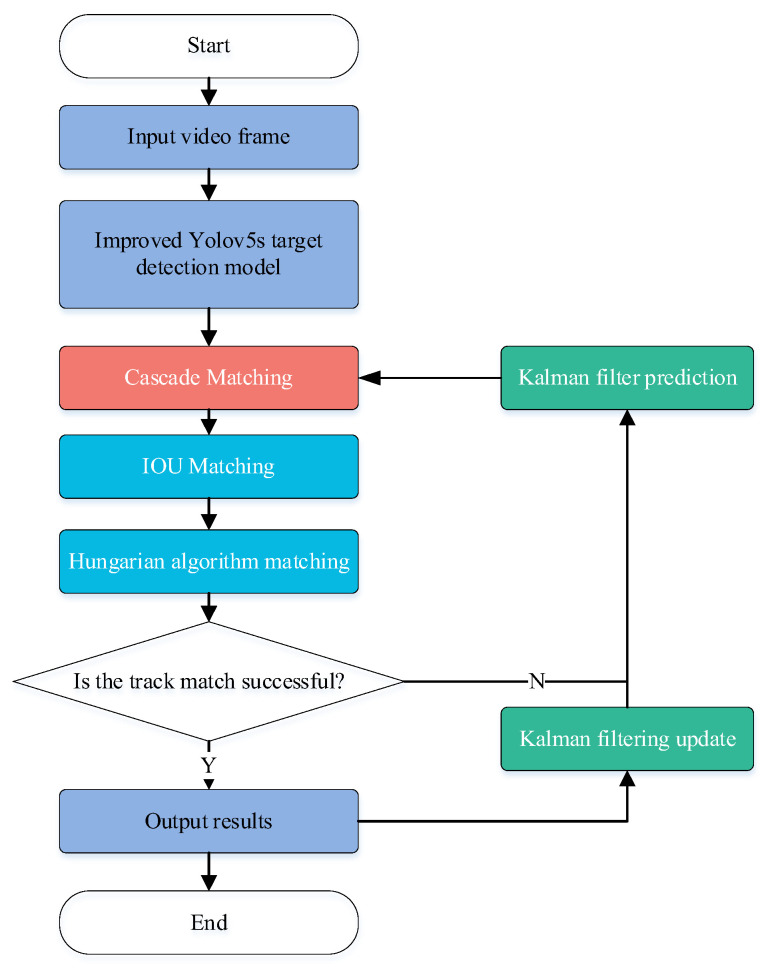
Flow chart of Tracking by Detecting.

**Figure 9:**
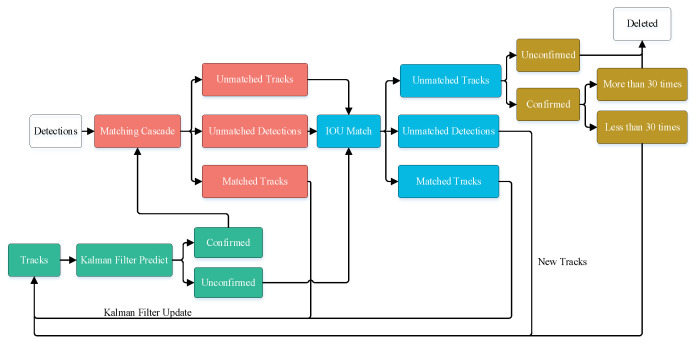
Flow chart of DeepSort tracking.

The detection result output of the improved YOLOv5s target detection algorithm was used as the input to the DeepSort algorithm, and the algorithm is illustrated in Figure 8. The detailed structure of the DeepSort algorithm is shown in Figure 9.

### 2.3. Model Training Environment and Parameter Settings

The model was run on a supercomputer at the Beijing Super Cloud Computing Center (Beilong Super Cloud Computing Co., Ltd., Beijing, China). The supercomputer was a Linux CentOS 7.9 and had a CPU consisting of two AMD EPYC 7282 16-core 32-thread processors, a RAM memory of 256 GB, and a NVIDIA RTX3090 graphics card with 24 GB video memory. The model was run in the software environment of NVIDIA CUDA11.3, torch 0.10.0, and Python3.8.

The training parameter settings for each ablation experiment were consistent. The input network image was 640 × 640 pixels with batch size set to 16, an initial learning rate of 0.001, and iterations of 100, and the model data were saved for every other iteration.

### 2.4. Model Optimization and Assessment

A series of simulated experiments were conducted to compare the Yolov5 algorithm with and without the use of dilated convolution and to investigate the impact of different attention mechanisms on the algorithm performance. Based on the results of simulated experiments, a high-precision algorithm was selected and pruned at different scales, which ultimately led to an optimized model with high recognition speed and precision that could be easily deployed to embedded-edge computing nodes. The optimized model was also compared with other nano-algorithms to assess the model performance in terms of recall rate and mean average precision (mAP). The number of parameters, recognition time per frame and frames per second (FPS) were used to evaluate the smoothness of recognition and tracking algorithms. 

In addition, a 2 min video of three pigs (different videos of the same type as the training data) was viewed several times by three humans to record pig postures and the time budget. The human observations were then compared with the model’s results to verify the model’s performance.

### 2.5. Deployment to Embedded-Edge Computing Nodes

**Figure 10:**
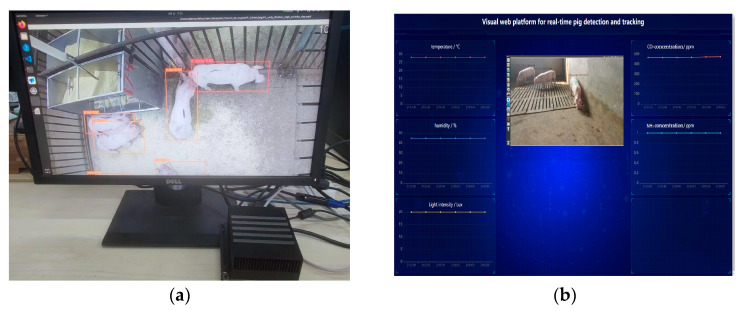
Jetson AGX Xavier embedded-edge computing node. (**a**) Edge computing node operation diagram; (**b**) Visual web platform diagram.

The embedded system hardware platform consisted of a Jetson AGX Xavier computer (NVIDIA Semiconductor Technology Co., Ltd., Shanghai, China) with NVIDIA Carmel ARMv8 64-bit CPU and 16 GB of running memory and a GPU with 512 NVIDIA CUDA cores and 64 Tensor Cores (Figure 10). The operating system was Ubuntu18.04 with ARM, NVIDIA CUDA 10.2, torch 0.10.0, python 3.8, and TensorRT 7.1.3. The Yolov5 and DeepSort runtime environments were installed and configured in the Jetson AGX Xavier edge computing node, and TensorRT C++ acceleration was implemented. The trained posture recognition models were ported to the edge computing node for field deployment. At the same time, the visual web platform could obtain the operation data of the edge nodes in real time and display it in the web browser.

## 3. Results and Discussion

### 3.1. Model Training with Different Modifications to YOLOv5s

#### 3.1.1. Effect of Dilated Convolution

Compared with the original model, the model with added dilated convolution resulted in a slight increase from 95.2% to 95.8% in precision and a slight decline in recall rate (from 96.6% to 96.3%). The mean average precision (mAP) remained unchanged at 97.8%. These slight changes were because the prediction results were screened strictly during training, which removed some positive examples with low confidence, resulting in a slight reduction in the recall rate, but the value was still greater than 95%, which is considered to be acceptable for practical applications.

#### 3.1.2. Effect of Adding Different Attention Mechanisms

Generally speaking, adding an attention mechanism should bring about an increase in the precision and a decrease in the recall rate [28]. Compared with the convolutional block attention module (CBAM) and Squeeze-and-Excitation Networks (SEs), the coordinate attention [10] mechanism increased the precision by 0.7% while the mAP remained almost the same (Table 2). In other words, adding the CA mechanism made the model slightly more sensitive.

#### 3.1.3. Overall Comparison Model Performance in Training

**Figure 11:**
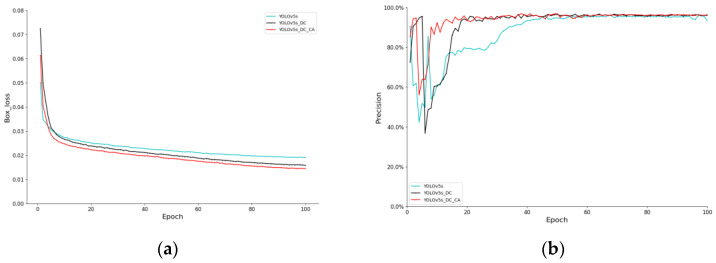
(**a**) The box losses of different models; (**b**) The training precision of three models.

Three models, namely, the original YOLOv5s, YOLOv5s with dilated convolution (YOLOv5s_DC), and YOLOv5s with both dilated convolution and CA attention (YOLOv5s_DC_CA), were compared for their training performance. For all three models, the loss curves decreased steadily and YOLOv5s_DC_CA had lower box loss at the end of training (at 100 epoch) (Figure 11a). The precision increased to a maximum level of about 96% for all three models, but YOLOv5s_DC_CA reached the maximum earlier than the other two models (Figure 11b). In other words, YOLOv5s with both dilated convolution and CA attention performed the best.

#### 3.1.4. Effect of Pruning Ratio

As shown in Table 3, with the increase in the pruning ratio of the BN layer for model YOLOv5s_DC_CA, the recall rate and the number of model parameters (model size) decreased correspondingly, while mAP fluctuated. The overall results indicated that the model of YOLOv5s_DC_CA at a pruning of 60% performed the best. This optimal model was 65.3% smaller than that of the un-pruning model, while the precision remained unchanged (mAP was only 0.7% points lower). When pruning was continued to 70%, the precision and recall rate decreased significantly compared with 60% pruning: specifically, there was a 1.9% and 2.8% reduction in the precision and recall rate, respectively. Therefore, the YOLOv5s_DC_CA model with a pruning rate of 60% was considered the optimal model and was used in the subsequent experiments.

#### 3.1.5. Recognition Performance for Different Pig Postures

The performance of the optimal model (YOLOv5s_DC_CA 60% Pruning rate) in recognizing four different pig postures is summarized in Table 4. The highest precision was recognizing the lying posture (98.6%) and the lowest was for the lateral (92.4%). The relatively low recognition precision for the lateral posture was mainly due to limb occlusion and overlap at some angles. The average precision of posture recognition was 96.6% and the average recall rate was 95.4%. This model’s performance is considered good and can meet the needs of practical applications [29]. 

#### 3.1.6. Comparisons with Other Models

Given that the primary goal of this research was to develop a high-performance model with a suitable (small) size for edge computing, two comparisons were conducted: one with high-performance models, and one with small-size models (Table 5). It was observed that the current model had similar precision and recall to the tested high-performance models (original YOLOv5s, YOLOX_s and PP_YOLOv3) but was much smaller in size. In comparison with two small-size models (YOLOX_nano and PP_YOLOTiny), the current mode was slightly larger but had better performance in terms of precision and recall (Table 5).

### 3.2. Performance of Model at Edge Computing Node (EECN)

The ultimate goal of this research was to develop the most suitable model to be deployed in an embedded-edge computing node (EECN) for the real-time identification and tracking of pigs in barns. Therefore, further tests were conducted to evaluate the model performance in the EECN environment.

#### 3.2.1. Target Detection

The first set of tests was performed to evaluate the effect of the pruning ratio on the performance of the YOLOv5s_DC_CA model. With the increase of the pruning ratio, the number of model parameters decreased rapidly, and the recognition time decreased gradually, while the frame rate increased (Table 6).

Combining the observations in Table 3 and Table 6 shows: (i) the YOLOv5s_DC_CA 60% Pruning rate could achieve 95% or higher precision and mAP; (ii) the number of model parameters was only 33% of the original YOLOv5s; (iii) the single frame inference time was reduced by 19.3%; and the recognition frame rate increased from 32.2 to 40.0 frames. This frame rate is suitable for use in embedded-edge nodes in real-time stable operation, which is critical for the subsequent multi-target tracking processing of pigs in embedded-edge nodes and providing fast and stable input results.

#### 3.2.2. Multi-Target Tracking

With the increase in the model pruning ratio, the tracking time per frame gradually decreased, and the tracking frame rate increased steadily (Table 7). Compared with the original YOLOv5s combined with the DeepSort algorithm, the current model (YOLOv5s_DC_CA 60% Pruning rate with the DeepSort algorithm) reduced the tracking time per frame by 39% (0.075 s to 0.046 s). Also, the frame rate increased by 63% (from 13.3 to 21.7 FPS), indicating that the current model could run quickly and stably in real-time in the embedded-edge nodes. From the practical point of view, a higher frame rate (e.g., 21.7 FPS) results in smoother videos than a lower frame rate (e.g., 16 FPS), and more importantly, tracking more frames per second would reduce the tracking ID loss and jump caused by the speed problem during the tracking process.

**Figure 12:**
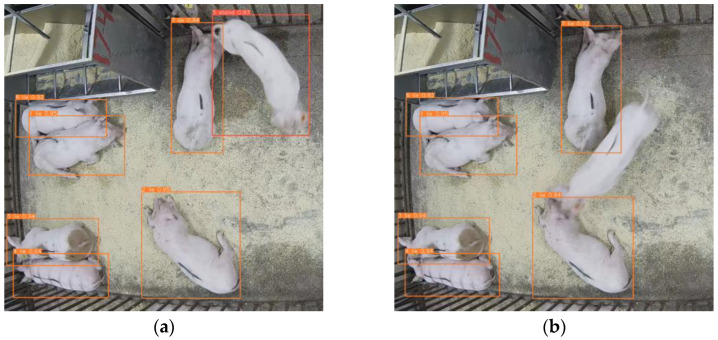

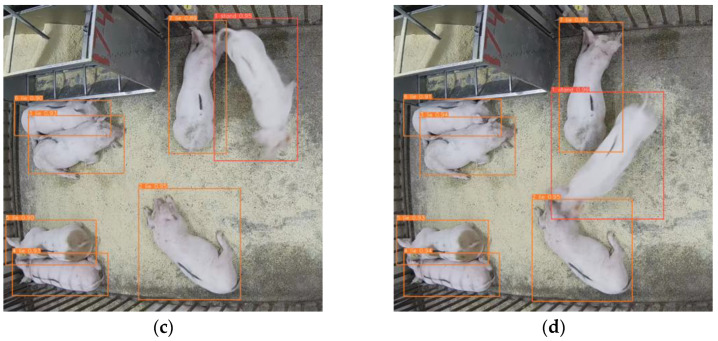
Screen shots of tracking pigs moving in pens by models running in embedded-edge computing nodes. (**a**) YOLOv5s + DeepSort for slight pig movement; (**b**) YOLOv5s + DeepSort for fast pig movement; (**c**) YOLOv5s_DC_CA 60% Pruning rate + DeepSort for slight pig movement; (**d**) YOLOv5s_DC_CA 60% Pruning rate + DeepSort for fast pig movement.

The trained tracking models were tested on three 60 s video clips of each with 3, 7 and 14 pigs in different postures. In comparison with the original YOLOv5s + DeepSort, the current model had higher tracking frame rate for all three scenarios, a lower number of ID jumps, and a lower number of missing tracking frames (Table 8). An important factor that affected the tracking performance of models was animal movement. When pigs moved little or slowly, the differences in performance between the original and the current models were not noticeable (Figure 12a,c). However, when pigs moved fast, the original YOLOv5 + DeepSort lost tracking (Figure 12b), whereas the current model was capable of carrying out stable tracking (Figure 12d).

**Figure 13:**
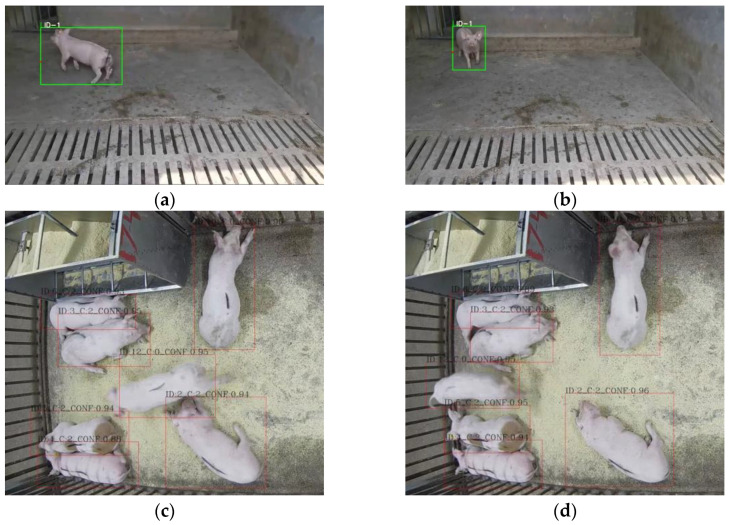

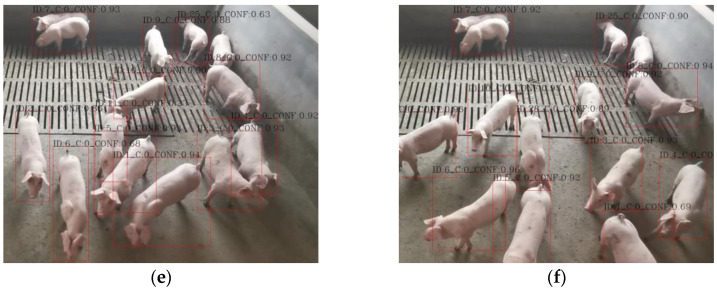
Real-time tracking results of pigs in different group sizes. (**a**) The 75th frame tracking results of 1 pig (side view); (**b**) The 130th frame tracking results of 1 pig (side view); (**c**) The 200th frame tracking results of 7 pigs (top view); (**d**) The 294th frame tracking results of 7 pigs (top view); (**e**) The 500th frame tracking results of 14 pigs; (**f**) The 500th frame tracking results of 14 pigs.

Further verification was conducted to evaluate the effect of the current model on the embedded-edge device for various group sizes (1, 7 and 15 pigs) with different camera view angles. From Figure 13, it can be seen that the proposed YOLOv5s_DC_CA 60% Pruning rate + DeepSort algorithm could properly detect and track pigs from different angles in different pig group sizes.

One of the important applications of the recognition and tracking model was to characterize pig behavior in real time by running the model in the embedded-edge computing node. This model functionality was tested on a 2 min clip of an unobstructed video of three pigs; specifically, pig postures and time budgets were quantified, and the results were compared with human observations. The algorithm had an overall agreement rate with human observations greater than 90% (Table 9). 

## 4. Conclusions

The YOLOv5s algorithm was shown as a solid base to develop a lightweight algorithm for edge node deployment to detect and track pigs with video cameras in pig barns, and ultimately to assess pig behavior in real time. Various additions and optimizations were applied to the original YOLOv5s algorithm, and it was found that adding dilated convolution and coordinate attention and 60% pruning resulted in the best (optimal) algorithm—YOLOv5s_DC_CA 60% Pruning rate—in terms of model size and recall rate and precision. When deployed to an edge node (NVIDIA TensorRT C++ hardware acceleration), the frame rate reached 40 FPS (frames per second) on average and up to 111 FPS, indicating that the algorithm may provide a fast, stable, and highly precise real-time recognition of group-housed pigs. When combined with the DeepSort tracking algorithm and deployed to an edge node Jetson AXG Xavier, the algorithm (model) had a frame rate of 21.7 FPS, which meets the requirement for the stable real-time tracking of group-housed pigs in barns. The assessments of pig behavior (postures and time budgets) by the algorithm were in good agreement (>90%) with human observations. Overall, the algorithm may provide a foundation for an image-based system for monitoring and assessing the behavior of group-housed pigs.

## Figures and Tables

**Table 1:** Definition of the four types of pig posture.

Posture	Definition
Standing	body is supported by all four limbs
Sitting	front feet extended and hind feet with hips on the ground to support the body
Lying	all four limbs and abdomen on the ground
Lateral	one side of body on the ground

**Table 2:** Comparative results of different attention methods.

Models	Recall	Precision	mAP:0.5
YOLOv5s_DC_CBAM	94.7%	96.5%	97.1%
YOLOv5s_DC_SE	92.1%	96.0%	96.4%
YOLOv5s_DC_CA	95.9%	96.5%	97.7%

**Table 3:** Effect of pruning ratio on model performance and size.

Pruning Rate	Recall	Precision	mAP:0.5	Model Size
0%	95.9%	96.5%	97.7%	13.70 MB
30%	95.3%	95.8%	96.3%	8.98 MB
40%	95.0%	96.2%	96.5%	7.63 MB
50%	95.0%	96.2%	96.8%	6.17 MB
60%	95.4%	96.6%	97.0%	4.74 MB
70%	92.6%	94.5%	96.3%	3.86 MB
80%	BN layer is not sparse enough to prune			

**Table 4:** Recognition effect of different postures of pigs.

Posture	Recall	Precision	mAP:0.5
Average	95.4%	96.6%	97.0%
Standing	98.2%	98.2%	99.2%
Lying	98.1%	98.6%	99.3%
Siting	88%	95.7%	91.3%
Lateral	97.4%	92.4%	97.7%

**Table 5:** Comparison of different models.

Models	Recall	Precision	mAP:0.5	Model Size
YOLOv5s original version	96.6%	95.2%	97.8%	14.70 MB
YOLOX_s	94.8%	97.6%	97.8%	34.30 MB
YOLOX_nano	88.02%	95.47%	95.0%	3.70 MB
PP_YOLOv3	95.50%	93.80%	91.70%	234.00 MB
PP_YOLOTiny	90.06%	83.05%	85.56%	3.87 MB
YOLOv5s_DC_CA 60% Pruning rate	95.4%	96.6%	97.0%	4.74 MB

**Table 6:** Detection performance of different pruning ratios in embedded-edge computing nodes.

Models	Pruning Rate	Number of Model Parameters	Recognition Time per Frame (s)	Recognition Frame Rate (FPS)
YOLOv5s original version	0%	7,020,913	0.031	32.2
YOLOv5s_DC_CA	0%	7,043,761	0.033	30.0
YOLOv5s_DC_CA	30%	4,544,301	0.031	32.2
YOLOv5s_DC_CA	40%	3,843,219	0.029	34.5
YOLOv5s_DC_CA	50%	3,079,080	0.027	37.0
YOLOv5s_DC_CA	60%	2,310,245	0.025	40.0
YOLOv5s_DC_CA	70%	1,868,441	0.024	41.0

**Table 7:** Tracking performance of different pruning ratios in edge computing node environment.

Models	Tracking Time Per Frame (s)	Tracking Frame Rate (FPS)
YOLO5s + DeepSort	0.075	13.3
YOLOv5s_DC_CA + DeepSort	0.078	12.8
YOLOv5s_DC_CA 30% Pruning rate + DeepSort	0.066	15.1
YOLOv5s_DC_CA 40% Pruning rate + DeepSort	0.057	17.5
YOLOv5s_DC_CA 50% Pruning rate + DeepSort	0.047	21.2
YOLOv5s_DC_CA 60% Pruning rate + DeepSort	0.046	21.7
YOLOv5s_DC_CA 70% Pruning rate + DeepSort	0.047	21.2

**Table 8:** Comparison of tracking performance for three different scenarios.

Model	No. of Pigs in 30 s Video	Tracking Frame Rate (FPS)	Number of Missing Tracking Frames	Number of ID Jumps
YOLOv5s + DeepSort	3	16.7	3	1
YOLOv5s_DC_CA 60% Pruning rate + DeepSort	3	21.8	2	1
YOLOv5s + DeepSort	7	15.9	5	3
YOLOv5s_DC_CA 60% Pruning rate + DeepSort	7	21.0	3	2
YOLOv5s + DeepSort	14	15.5	20	13
YOLOv5s_DC_CA 60% Pruning rate + DeepSort	14	20.7	16	9

**Table 9:** Comparisons of algorithm assessment of pig behavior with human observations for three pigs in a pen.

Pig ID	Standing Time (s)	Sitting Time (s)	Lying Time (s)	Lateral Time (s)
Model, Pig 1	109	5	3	3
Human observation, Pig 1	118	2	0	0
Model, Pig 2	74	0	30	16
Human observation, Pig 2	75	0	34	11
Model, Pig 3	0	5	104	11
Human observation, Pig 13	0	0	110	10

## Data Availability

Not applicable.
